# It is premature to regard the ego-depletion effect as “Too Incredible”

**DOI:** 10.3389/fpsyg.2014.00298

**Published:** 2014-04-10

**Authors:** Martin S. Hagger, Nikos L. D. Chatzisarantis

**Affiliations:** Health Psychology and Behavioural Medicine Research Group, Laboratory of Self-Regulation, Faculty of Health Sciences, School of Psychology and Speech Pathology, Curtin UniversityPerth, WA, Australia

**Keywords:** self-regulation, self-control, strength model, meta-analysis, publication bias, incredibility index

The “strength” model conceptualizes self-control as a limited resource (Baumeister et al., 1998). Individuals are able to exert self-control, but only for a limited period after which capacity declines leading to reduced self-control capacity; a state known as *ego-depletion*. The model has generated a sizable literature confirming the ego-depletion effect in multiple spheres. Our meta-analysis of published ego-depletion studies computed a medium-sized effect (*d* = 0.62) across 198 tests (Hagger et al., 2010).

Carter and McCullough (2013) recently applied analyses aimed at testing for publication bias to our data including Schimmack's (2012) incredibility index and two regression techniques (Egger et al., 1997; Moreno et al., 2009). Regression analyses indicated that the ego-depletion effect was substantially smaller than reported in our analysis and may even be zero, and the incredibility index indicated low statistical power and the chances of finding so many significant effects improbable. They concluded that the ego-depletion effect is subject to considerable publication bias and questioned whether it is a “real” effect at all. We replicated these analyses and found similar results. We have made our analyses available to download from the open-access Dryad Digital Repository (Hagger and Chatzisarantis, 2014).

We thank Carter and McCullough (2013) raising the issue of bias. We take this opportunity to present some alternative conclusions to the ones they presented. We agree that journal editors should be more judicious in demanding bias tests in meta-analyses, but believe that that recommendation does not resolve the problem of *interpreting* the bias. An important addendum to the regression analyses is that the bias detected by a significant regression line cannot be definitively attributed to publication bias. Sterne et al. (2000, 2001) suggest that such bias could be attributed to a number of possible sources. Instead, they use the term “small study” effect; the tendency for smaller studies to report larger effect sizes. One possible reason would be due to publication bias: journals tending to favor the publication of small studies with statistically significant results and disproportionately large effect sizes. However, the findings may also be due to methodological inadequacies or true heterogeneity in the effect. A definitive response to resolving the nature of bias detected by these methods (i.e., whether it is publication bias or other source of bias that causes a “small study effect”) would be to demand authors conducting meta-analyses be diligent in the pursuit of “fugitive literature”: unpublished studies with null findings, or findings that conflict with the commonly-accepted paradigm, that Rosenthal (1994) eloquently predicted would reside in the “file drawers” of researchers who could not get them published. In the case of ego-depletion, a unique contribution would be to identify unpublished studies including those with null or negative effects, as well as studies that have since been published, and recalculate the meta-analytic effect size. Such an undertaking would not only yield a more robust effect size ostensibly independent of publication bias but also be informative as to whether the “small study effect” detected in the analyses was due to publication bias, other forms of bias, or true heterogeneity. We encourage researchers to make their replications of ego-depletion studies freely available to aid future meta-analyses.

We would also like to express concerns regarding Carter and McCullough's prediction, based on their regression analyses, that the ego-depletion effect may be zero. This prediction was based on the intercept of the regression of the ego-depletion effect size on precision. However, if the true ego-depletion effect size is zero or close to it, one would expect the effect sizes in the literature to be randomly distributed in both positive and negative directions about zero. If this is the case, then where are those negative findings? There are scant few ego-depletion experiments that have found opposite effects, i.e., an improvement in second-task performance after engaging in an initial self-control task, let alone null effects. Given the intensiveness of research in this field, would it not be reasonable to expect to have seen the negative findings published? The absence of these effects creates a problem for the claim that the true effect is zero and the credibility of the analysis.

It could be argued that such negative effects might not have been published because their interpretation might contradict commonly-accepted theory and may lie in the file drawers of the researchers who found them. However, we think that such findings would likely have seen the light of day in journals because they contradict the strength model and support alternative hypotheses consistent with other theories such as adaptation or learned industriousness (Converse and DeShon, 2009). For example, one could pose an alternative hypothesis that improvement in self-control performance in ego-depletion experiments could be due to learning the capacity to self-regulate which was transferable. In such cases one would expect statistically-significant improvements in performance on a subsequent self-control task after engaging in an initial task that taxes self-control. Of course, we would have to assume that researchers were sufficiently virtuous in not turning their null results into supportive evidence using selective reporting (Francis, 2014). We contend that if the predicted effect size for ego-depletion is zero, then negative effects should be present in this literature and we would expect such effects to be published given their pivotal role in testing alternative hypotheses based on other theories.

As a final point, while we thank Carter and McCullough for raising a notable question regarding the existence of bias in ego-depletion meta-analysis, their analysis tells us little about its source and does not acknowledge that other effects in published meta-analyses are subject to similar bias. We think it is important to view and interpret the bias found for ego-depletion using these methods in context. For example, how does the small study effect found for ego depletion match up to the relative to the incidence of bias in the discipline of social psychology as whole? A useful future endeavor would be to systematically identify meta-analyses published in social psychology over a substantive period, subject each to the bias-identification analyses, and comment on the extent of the bias within the discipline.

## Author contributions

Martin S. Hagger and Nikos L. D. Chatzisarantis conceived the ideas presented in the article and drafted the article.

## Conflict of interest statement

The authors declare that the research was conducted in the absence of any commercial or financial relationships that could be construed as a potential conflict of interest.

## Supplementary material

The Supplementary Material for this article can be found online at: http://www.frontiersin.org/journal/10.3389/fpsyg.2014.00298/abstract

Figure S1 **Funnel plot of the ego-depletion effect size (standardized mean difference, Cohen's d) against the against the study precision (1/standard error)**.

Figure S2 **Funnel plot of the ego-depletion effect size (standardized mean difference, Cohen's d) against the standard error of the effect size**.
