# Supplementary material for: It is premature to regard the ego-depletion effect as “Too Incredible”
Source: Front Psychol. 2014 Apr 10;5:298. doi: 10.3389/fpsyg.2014.00298 (PMC3989757; doi:10.3389/fpsyg.2014.00298)
Supplement: Supplementary file 3 [file DataSheet3.DOCX]

**Introduction to publication bias**

The basic issue of publication bias is that not all completed studies are published, and the selection process is not random (hence the 'bias'). Rather, studies that report relatively large treatment effects are more likely to be submitted and/or accepted for publication than studies which report more modest treatment effects.

Since the treatment effect estimated from a biased collection of studies would tend to overestimate the true treatment effect, it is important to assess the likley extent of the bias, and its potential impact on the conclusions. This module includes several tools which may be used for this purpose.

**Funnel plot**

The funnel plot is a plot of a measure of study size (usually standard error or precision) on the vertical axis as a function of effect size on the horizontal axis.

Large studies appear toward the top of the graph, and tend to cluster near the mean effect size. Smaller studies appear toward the bottom of the graph, and (since there is more sampling variation in effect size estimates in the smaller studies) will be dispersed across a range of values.

In the absence of publication bias we would expect the studies to be distributed symmetrically about the combined effect size. By contrast, in the presence of bias, we would expect that the bottom of the plot would show a higher concentration of studies on one side of the mean than the other. This would reflect the fact that smaller studies (which appear toward the bottom) are more likely to be published if they have larger than average effects, which makes them more likely to meet the criterion for statistical significance.

Various statistical procedures can be accessed from the View menu to quantify or augment this display.

The classic fail-Safe N and the Orwin fail-safe N ask if we need to be concerned that the entire observed effect may be an artifact of bias. Rank correlation and regression procedures can test for the presence of bias. Trim and Fill offers a more nuanced perspective, and asks how the effect size would shift if the apparent bias were to be removed.

**Classic fail-safe N**

One concern of publication bias is that some non-significant studies are missing from our analysis and that these studies, if included, would nullify the observed effect.

Robert Rosenthal suggested that rather than simply speculate about the impact of the missing studies, we compute the number of studies that would be required to nullify the effect. If this number is relatively small then there is indeed cause for concern. However, if this number is large, we can be confident that the treatment effect, while possibly inflated by the exclusion of some studies, is nevertheless not nil.

He suggested that this analysis be called a 'File-drawer' analysis, file drawers being the presumed location of the missing studies. Harris Cooper proposed the term 'Fail-Safe N', a reference to the number of missing studies that would nullify the effect.

This approach is limited in two important ways. First, it assumes that the effect in the hidden studies is nil, rather than considering the possibility that some of the studies could have shown an effect in the reverse direction. Therefore, the number of studies required to nullify the effect may be smaller than the Fail-Safe N.

Second, and more fundamentally, this approach focuses on statistical significance rather than clinical or substantive significance. That is, it may allow us to assert that the treatment effect is not nil, but does not address the question of whether or not it remains clinically important after the missing studies have been included.

Note also that the fail-safe N algorithm computes a p-value for each study and then combines these p-values. By contrast, the generally accepted approach today (and the one used by this program) is to compute an effect size for each study, combine the effect sizes, and then compute the p-value for the combined effect. The two approaches do not generally yield identical results.

This meta analysis incorporates data from 198 studies, which yield a z-value of 33.21200 and corresponding 2-tailed p-value of 0.00000.

The fail-safe N is 56656. This means that we would need to locate and include 56656 'null' studies in order for the combined 2-tailed p-value to exceed 0.050.

Put another way, there would be need to be 286.1 missing studies for every observed study for the effect to be nullified.

**Orwin fail-safe N**

Like the classic fail-safe N, the Orwin fail-safe N addresses the possibility that studies are missing from the analysis and that these studies, if included in the analysis, would shift the effect size toward the null.

Orwin's fail-safe N differs from the classic fail-safe N in two ways.

First, the mean std diff in means in the new (missing) studies can be a value other than the nil value (currently, it is set to 0).

Second, the criterion value is an effect size rather than a p-value. That is, the Orwin fail-safe N is the number of (missing) studies that, when added to the analysis, will move the combined std diff in means past a specified threshold (currently, 0).

The criterion value must be set between the other two values for the Orwin fail-safe N to be computed.

**Begg and Mazumdar Rank Correlation Test**

The classic case of publication bias is the case depicted by the funnel plot. Large studies tend to be included in the analysis regardless of their treatment effect whereas small studies are more likely to be included when they show a relatively large treatment effect. Under these circumstances there will be an inverse correlation between study size and effect size.

Begg and Mazumdar suggested that this correlation can serve as a test for publication bias. Concretely, they suggest that we compute the rank order correlation (Kendall's tau b) between the treatment effect and the standard error (which is driven primarily by sample size).

This approach is limited in some important ways. A significant correlation suggests that bias exists but does not directly address the implications of this bias. Conversely, a non-significant correlation may be due to low statistical power, and cannot be taken as evidence that bias is absent.

In this case Kendall's tau b (corrected for ties, if any) is 0.43336, with a 1-tailed p-value (recommended) of 0.00000 or a 2-tailed p-value of 0.00000 (based on continuity-corrected normal approximation).

**Egger's Test of the Intercept**

Egger suggests that we assess this same bias by using precision (the inverse of the standard error) to predict the standardized effect (effect size divided by the standard error). In this equation, the size of the treatment effect is captured by the slope of the regression line (B1) while bias is captured by the intercept (B0).

This approach may offer a number of advantages over the rank correlation approach. Under some circumstances this may be a more powerful test. Additionally, this approach can be extended to include more than one predictor variable, which means that we can simultaneously assess the impact of several factors, including sample size, on the treatment effect.

In this case the intercept (B0) is 2.68241, 95% confidence interval (2.24619, 3.11863), with t=12.12704, df=196. The 1-tailed p-value (recommended) is 0.00000, and the 2-tailed p-value is 0.00000.

**Duval and Tweedie's Trim and Fill**

If the meta analysis had captured all the relevant studies we would expect the funnel plot to be symmetric. That is, we would expect studies to be dispersed equally on either side of the overall effect. Therefore, if the funnel plot is actually asymmetric, with a relatively high number of small studies (representing a large effect size) falling toward the right of the mean effect and relatively few falling toward the left, we are concerned that these left-hand studies may actually exist, and are missing from the analysis.

Duval and Tweedie developed a method that allows us to impute these studies. That is, we determine where the missing studies are likely to fall, add them to the analysis, and then recompute the combined effect.

The method is known as 'Trim and Fill' as the method initially trims the asymmetric studies from the right-hand side to locate the unbiased effect (in an iterative procedure), and then fills the plot by re-inserting the trimmed studies on the right as well as their imputed counterparts to the left the mean effect.

The program is looking for missing studies based on a fixed effect model, and is looking for missing studies only to the left side of the mean effect (these parameters are set by the user). Using these parameters the method suggests that 73 studies are missing.

Under the fixed effect model the point estimate and 95% confidence interval for the combined studies is 0.62257 (0.58351, 0.66163). Using Trim and Fill the imputed point estimate is 0.47769 (0.44250, 0.51288).

Under the random effects model the point estimate and 95% confidence interval for the combined studies is 0.67866 (0.62814, 0.72918). Using Trim and Fill the imputed point estimate is 0.50030 (0.44454, 0.55606).

To plot the imputed studies click 'Funnel plot' and then select 'Plot observed and imputed' on the toolbar.

**An important caveat**

Sterne and Egger note that while the plot and these procedures may detect a relationship between sample size and effect size, they cannot assign a causal mechanism to it.

That is, the effect size MAY be larger in small studies because we retrieved a biased sample of the smaller studies. However, it is also possible that the effect size really IS larger in smaller studies - perhaps because the smaller studies used different populations or different protocols than the larger ones.

Sterne and Egger use the term 'small study effect' to capture these and other potential confounds.
