# Supplementary material for: It is premature to regard the ego-depletion effect as “Too Incredible”
Source: Front Psychol. 2014 Apr 10;5:298. doi: 10.3389/fpsyg.2014.00298 (PMC3989757; doi:10.3389/fpsyg.2014.00298)
Supplement: Supplementary file 2 [file DataSheet1.DOCX]

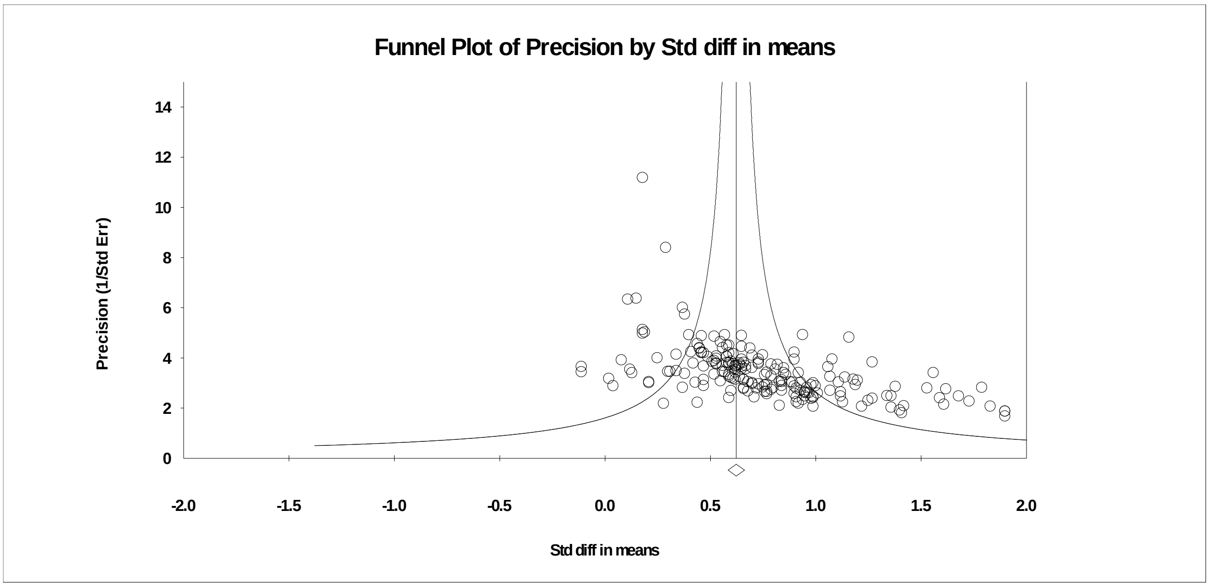
Funnel Plot of Precision by Std diff in means
Precision (1/Std Err)
14
12
10
8
6
4
2
0
-2.0
-1.5
-1.0
-0.5
0.0
0.5
1.0
1.5
2.0
Std diff in means
